# Supplementary material for: Electronic structure modulation of iron sites with fluorine coordination enables ultra-effective H2O2 activation
Source: Nat Commun. 2024 Mar 12;15:2241. doi: 10.1038/s41467-024-46653-6 (PMC10933296; doi:10.1038/s41467-024-46653-6)
Supplement: Supplementary file 1 — Supplementary Information [file 41467_2024_46653_MOESM1_ESM.pdf]

Supplementary Information

Electronic Structure Modulation of Iron Sites with Fluorine Coordination Enables Ultra-effective H_2O_2 Activation

Deyou Yu[1#], Licong Xu[1#], Kaixing Fu[2], Xia Liu[3], Shanli Wang[1], Minghua Wu[1], Wangyang Lu[4], Chunyu Lv[2], Jinming Luo[2]*

[1] Engineering Research Center for Eco-Dyeing and Finishing of Textiles (Ministry of Education), Zhejiang Sci-Tech University, Hangzhou 310018, PR China

[2] School of Environmental Science and Engineering, Shanghai Jiao Tong University, Shanghai 200240, PR China

[3] College of Chemistry and Chemical Engineering, Qingdao University, Qingdao 266071, PR China

[4] School of Material Science & Engineering, Zhejiang Sci-Tech University, Hangzhou 310018, PR China

[#]Deyou Yu and Licong Xu contributed equally to this work.

*Corresponding author: Jinming Luo (jinming.luo@sjtu.edu.cn).

This PDF file includes:

- Supplementary Notes 1 to 6
- Supplementary Figures 1 to 45
- Supplementary Tables 1 to 7
- Supplementary References 1 to 31

Supplementary Notes

Supplementary Note 1: Chemicals and Materials.

Fluorosilicic acid solution (30%), iron powder, n-propanol (NPA), Iron (III) chloride ($FeCl_3$•$6H_2O$), p-nitrophenol (4-NP), tert-Butanol (TBA), methanol (MeOH), acetonitrile (ACN), 4-amino-2,2,6,6-tetramethylpiperidine (TEMP), 5,5-dimethyl-1-pyrroline-N-oxide (DMPO), sodium sulfate (Na_2SO_4), coumarin (COU), and 7-hydroxycoumarin (7-HC) were purchased from Aladdin Bio-Tech Co., Ltd. Hydrogen peroxide (H_2O_2, 30%) was provided by Sinopharm Chemical Reagent Co., Ltd. Ethanol (EA), hydrochloric acid (HCl), sodium hydroxide (NaOH), and sodium chloride (NaCl) were obtained from Hangzhou Gaojing Fine Chemical Co., Ltd. Tetracycline, ofloxacin, atrazine, sulfamethoxazole were purchased from Macklin Biochemical Co., Ltd. All the chemical reagents were used as received without any further purification. The FeOCl catalyst was synthesized by heating $FeCl_3$•$6H_2O$ powder at a heating rate of 10 °C min^{-1} to 220 °C and annealing for 2 h.

Supplementary Note 2: Measurement procedure for and quantification details for Lewis acid sites.

The Py-IR spectra of prepared catalysts were recorded using a Thermo Nicolet iS10 FTIR spectrometer equipped with KBr windows. Prior to recording, the sample was pressed into a self-supporting wafer and activated in vacuum ($<10^{-4}$ Pa) at 200 °C for 1 h. For pyridine adsorption, the samples were exposed to pyridine for 1 h after cooling down, then the IR transmission cell was evacuated to vacuum. The spectra were collected at degassing temperatures of 50, 100, and 200 °C to verify the type of acid sites. For the quantitative comparison, the area of peak at 1445 cm^{-1} and a molar absorption coefficient of 1.42 cm μmol^{-1} were used to calculate the Lewis acidity, following the methodology established in previous reports (1-3).

Supplementary Note 3: Theoretical calculations.

The Vienna Ab initio Simulation Package (VASP) (4) was employed to perform all density functional theory (DFT) calculations with the generalized gradient approximation (GGA) using the Perdew-Burke-Ernzerhof (PBE) functional (5, 6). We have chosen the projected augmented wave (PAW) (7) potentials to describe the ionic cores and take valence electrons into account using a plane wave basis set with a kinetic energy cutoff of 500 eV. A 2×1 supercell was used on both for (110), (101), (211) surfaces and 2×2 supercell was used for the (010) surface, respectively. The bottom four atomic layers was fixed, while

atoms in other layers were fully relaxed during geometry optimizations. Monkhorst-Pack k-points of 5×5×1 was applied for all the surface calculations. All the structures were fully relaxed until the forces on each atom (except for those fixed ones) were less than 0.01 eV/Å and the convergence criterion for the electronic structure iteration was set to be 10^{-5} eV. The van der Waals (vdW) interactions were described by using the empirical correction in Grimme's method (DFT+D3) (8). The vacuum space was set to be 15 Å in the z direction, which was large enough to minimize the interactions between periodic images.

Supplementary Note 4: •OH measurement and quantification.

The •OH radicals were quantified using a fluorescence method with coumarin as the probe, which could be converted into 7-hydroxycoumarin (7-HC) with 29% selectivity. The 7-HC concentration was measured by a fluorescence spectrophotometer (Hitachi F-4600) with an emission wavelength of 456 nm and the excitation wavelength of 340 nm. We first plotted the standard curve of 7-hydroxyl coumarin concentration versus fluorescence intensity. The resultant equation is determined to be "Intensity = 37.7 × [7-HOC] (R^2 > 0.999)". Then, we measured the intensity of withdrawn samples, whose 7-hydroxyl coumarin (7-HOC) concentration could be easily determined by the obtained equation. Finally, we calculated the accumulated concentration of •OH by following equation.

$$[\bullet OH] = 2 \times [7\text{-HOC}]/Se$$

where [7-HOC] and Se indicate the concentration of 7-HOC and the selectivity, respectively.

The contribution of free and surface-bound •OH radicals were quantified and calculated by following equations according to previous study using the k_{obs} before and after quenching (9).

$$\theta_1 = [(k_{obs}^0 - k_{obs}^1)/k_{obs}^0] \times 100\%$$
$$\theta_2 = [(k_{obs}^0 - k_{obs}^2)/k_{obs}^0] \times 100\%$$
$$\theta_3 = \theta_1 - \theta_2$$

where the θ_1, θ_2, and θ_3 indicate the contribution ratio of total •OH, surface-bound •OH, and free •OH, respectively. The k_{obs}^0, k_{obs}^1, and k_{obs}^2 represent the apparent kinetic rate constant of 4-NP degradation by FeOF/H_2O_2 system with no quencher addition, TBA addition, and MeOH addition, respectively.

Supplementary Note 5: Antibacterial activity assessment.

E. coli (ATCC 25922, Shanghai Luwei Technology Co., Ltd.) was used as the model microorganism to test the antibacterial activity of FeOF catalyst. *E. coli* was grown in Luria−Bertani broth at 37 °C overnight, diluted in fresh medium, and washed three times

with a sterile saline solution (0.9% NaCl at pH 6.6) prior to further experiments. *E. coli* inactivation was assessed using the plate count method (10-12). Briefly, FeOF catalyst was added into a diluted bacteria suspension (10^7 colony forming unit (CFU) mL^{-1}) to achieve a final concentration of 0.1 g L^{-1}, followed by an immediate addition of H_2O_2 (1 mM) to initiate the Fenton reaction. After 2 min of reaction, the bacterial suspension was diluted by 10^3, 10^4, and 10^5-fold with sterilized water. The supernatant was spread on a nutrient agar medium and cultured at 37 °C for 24 h prior to enumeration. Control experiments were performed to evaluate the intrinsic antibacterial capacity of H_2O_2 alone.

Supplementary Note 6: Trials for wastewater treatment.

50 mg catalyst was first added into a 100-mL beaker containing 50 mL collected dyeing and printing wastewater or pharmaceutical wastewater. Subsequently, a certain amount of H_2O_2 (100 mM) was added to initiate the Fenton catalysis reaction due to the presence of large number of organic pollutants. After reaction for 30 min, 5 mL of treated wastewater was withdrawn and filtered through a syringe filter by 0.22 μm membrane for further analysis. The pH value, TOC and EEM spectra of the pre- and post-treatment wastewater were finally analyzed to evaluate the practicality of FeOF/H_2O_2 system. Effluent A, B, and C are kindly provided by Zhejiang Daneng Textile Co., Ltd., Hangzhou Huasi Xiasha Textile Technology Co., Ltd., and People's Hospital of Wangcheng District Changsha, respectively.

Supplementary Figures

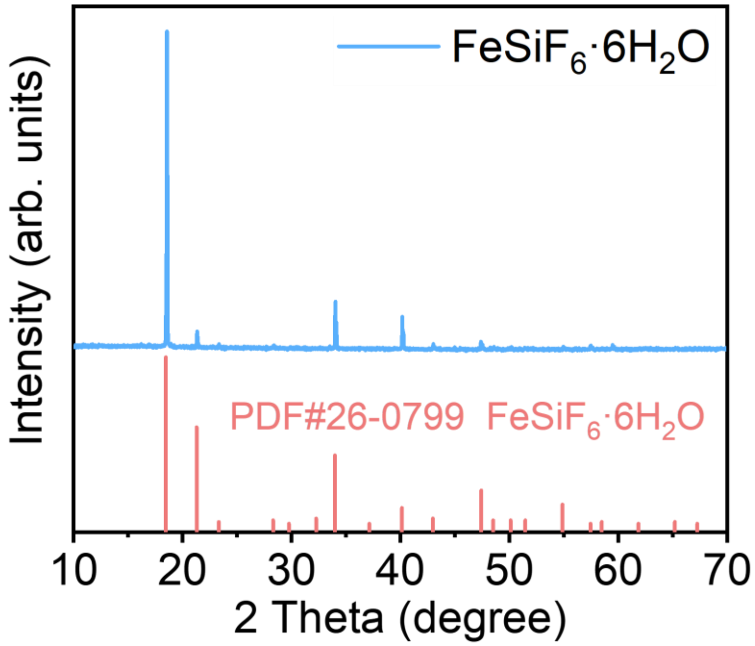

Supplementary Fig. 1. X-ray diffraction pattern of $FeSiF_6.6H_2O$.

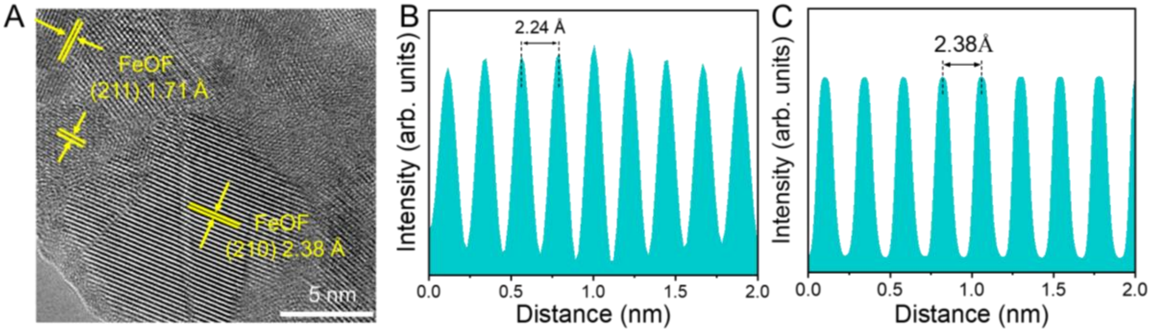

Supplementary Fig. 2. (A) HRTEM image of FeOF catalyst with highlights of (211) and (210) facets. (B) Intensity profile of (111) facet in Fig. 1C indicates a d spacing of 2.24 Å. (C) Intensity profile of (111) facet in Supplementary Fig. 2A indicates a d spacing of 2.38 Å for (210) facet.

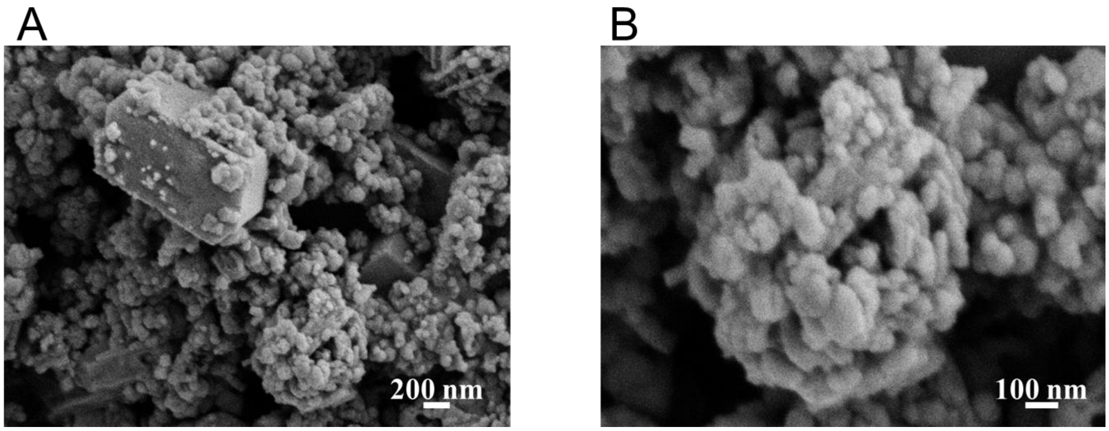

Supplementary Fig. 3. FE-SEM images of FeOF with relatively small (A) and large (B) magnifications.

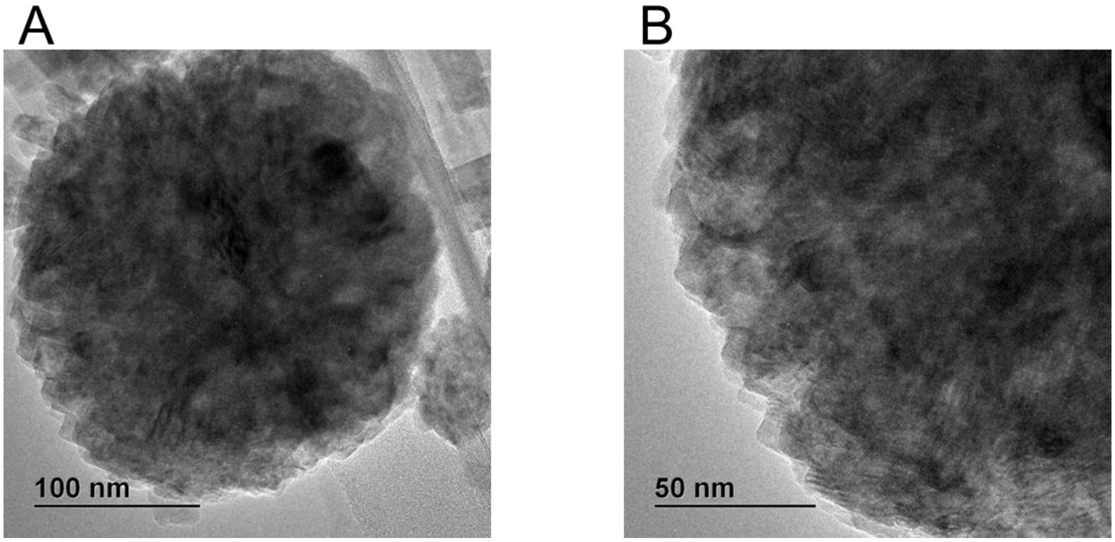

Supplementary Fig. 4. TEM images of FeOF with relatively small (A) and large (B) magnifications.

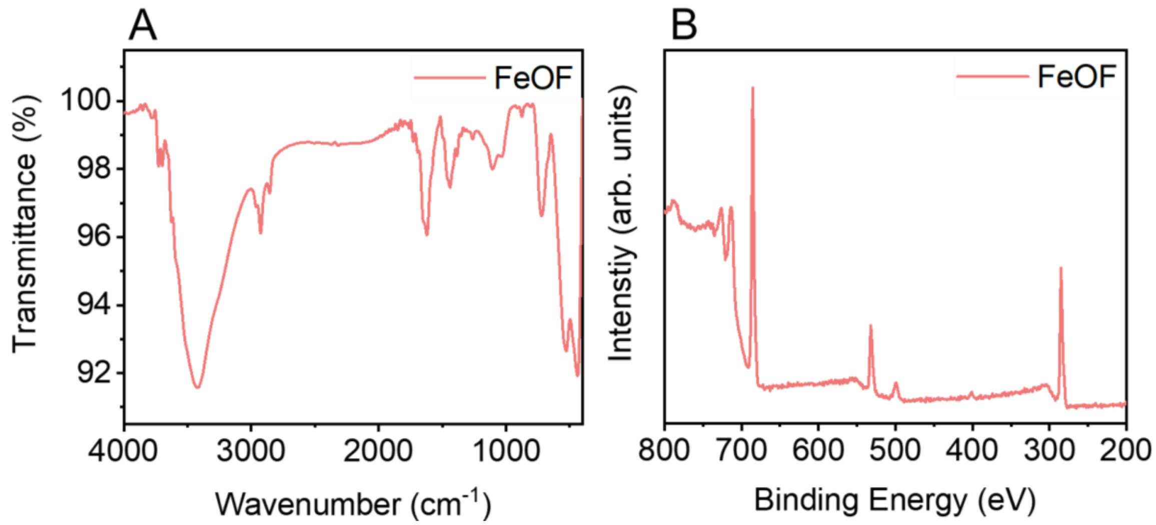

Supplementary Fig. 5. FT-IR spectrum (A) and XPS survey spectrum (B) of FeOF.

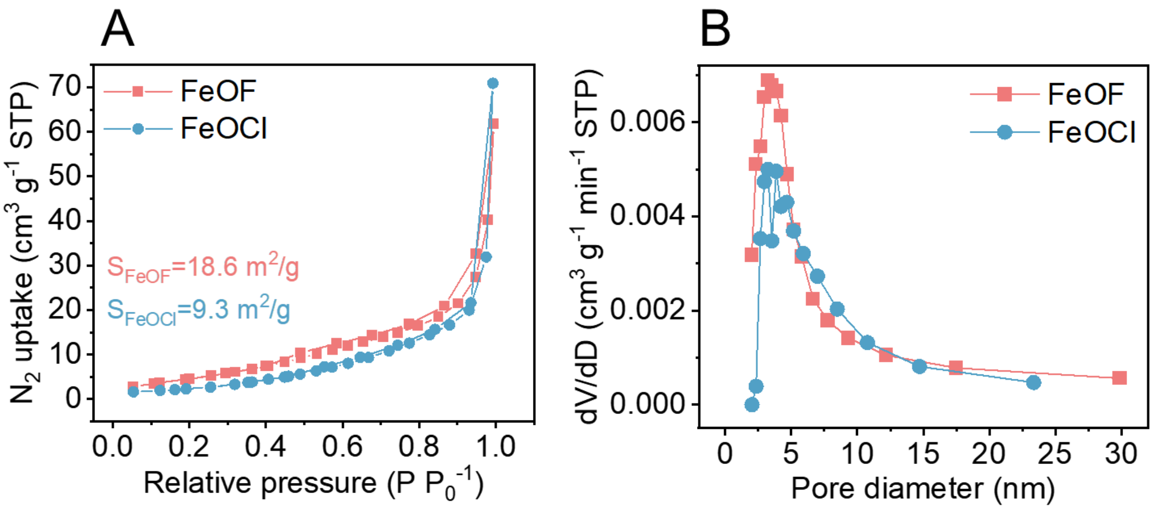

Supplementary Fig. 6. N_2 adsorption-desorption isotherms (A) and pore distributions (B) of FeOF and FeOCl.

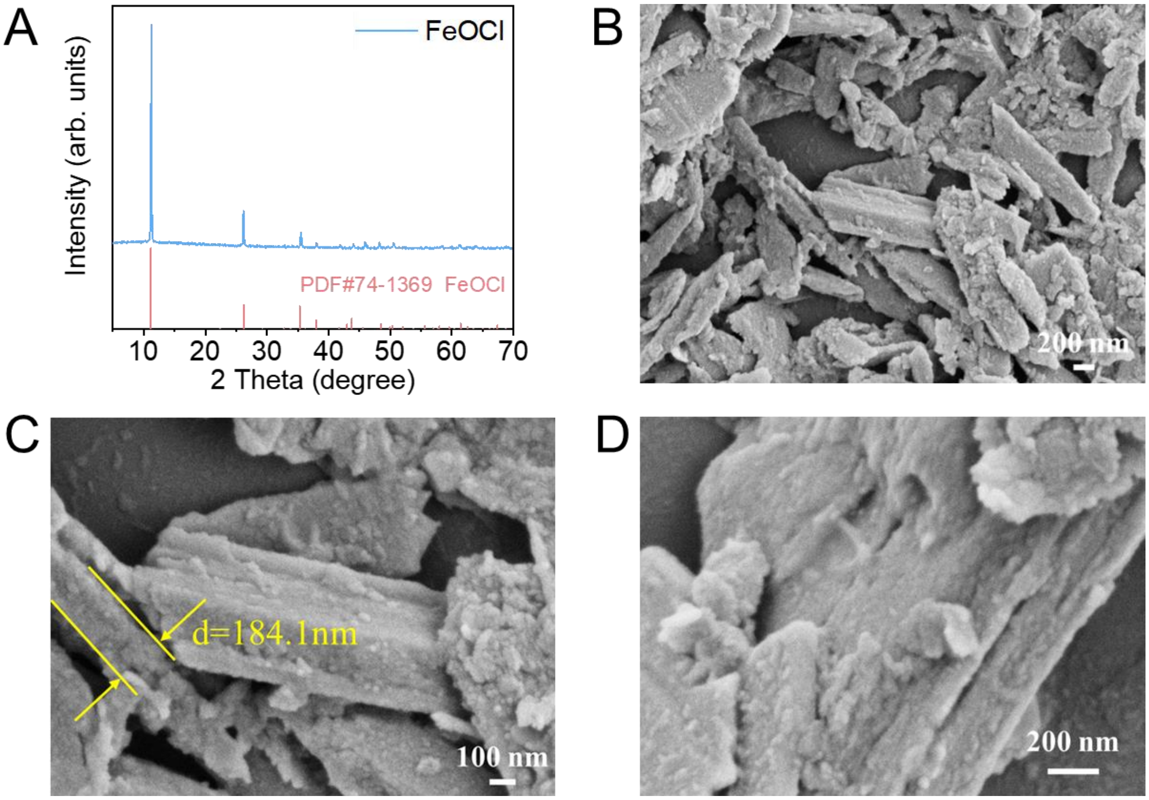

Supplementary Fig. 7. Characterization of FeOCl. (A) XRD pattern and (B-D) FE-SEM images.

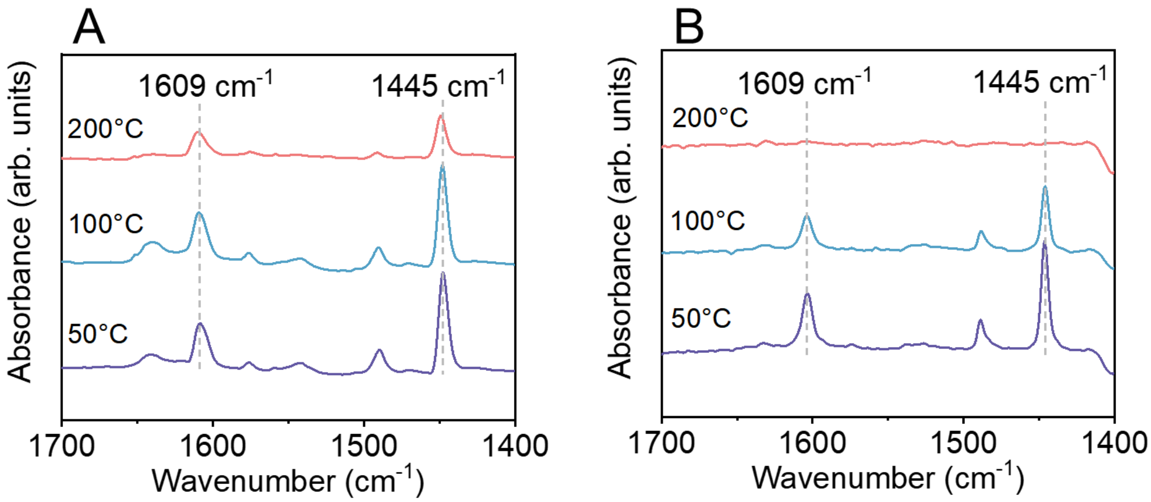

Supplementary Fig. 8. Py-IR spectra of FeOF (A) and FeOCl (B).

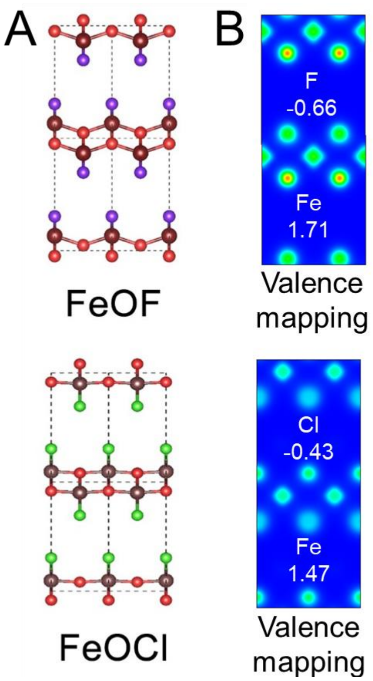

Supplementary Fig. 9. (A) Side view of the optimized molecular structure geometries for FeOF and FeOCl, in which the layer-by-layer architecture is well-recognized. The brown, red, violet, and green balls represent the Fe, O, F, and Cl atoms, respectively. (B) Corresponding two-dimensional valence-electron density color-filled maps. The simulated valence states of Fe, F, and Cl were highlighted in both plots. However, it is important to note that direct comparisons of valence states between different plots are not reasonable based solely on the colors displayed in the two diagrams.

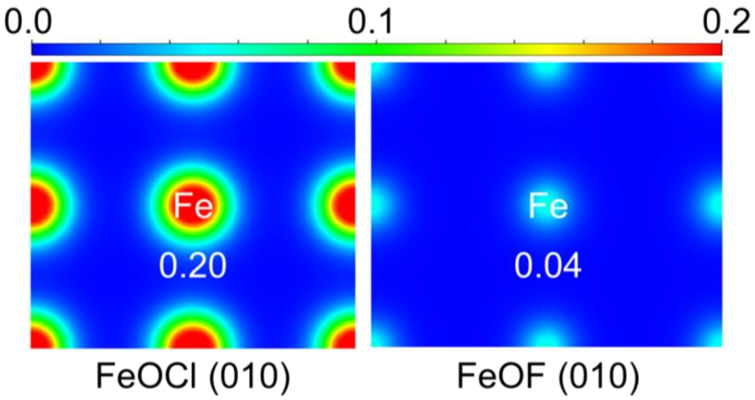

Supplementary Fig. 10. Maximum valence-electron density of iron sites in FeOF and FeOCl.

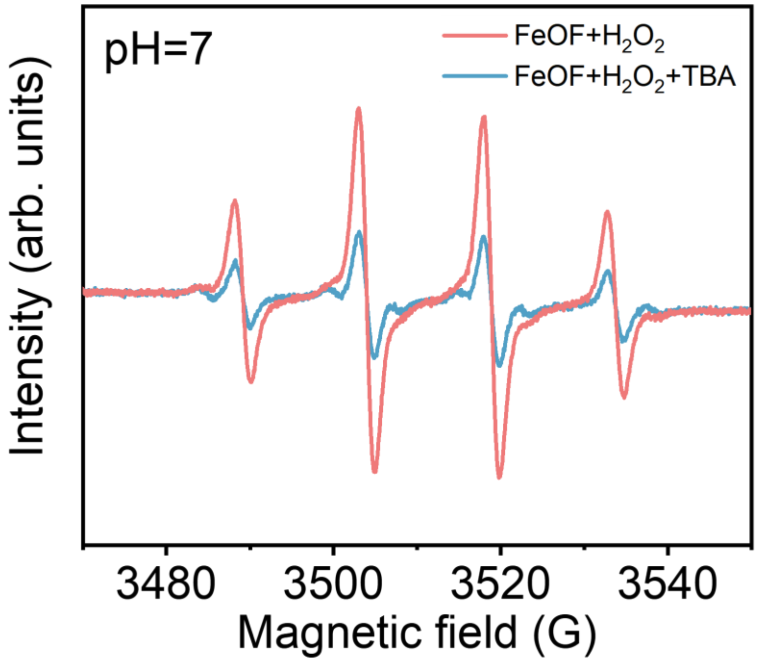

Supplementary Fig. 11. DMPO-•OH signals in EPR spectra of FeOF/H_2O_2 system with and without TBA addition. Reaction conditions: [H_2O_2] = 10 mM, [catalyst] = 1.0 g L^{-1}, [DMPO] = 10 mM, [TBA] = 10 mM (if used), pH = 7.0, Temperature = 20 °C.

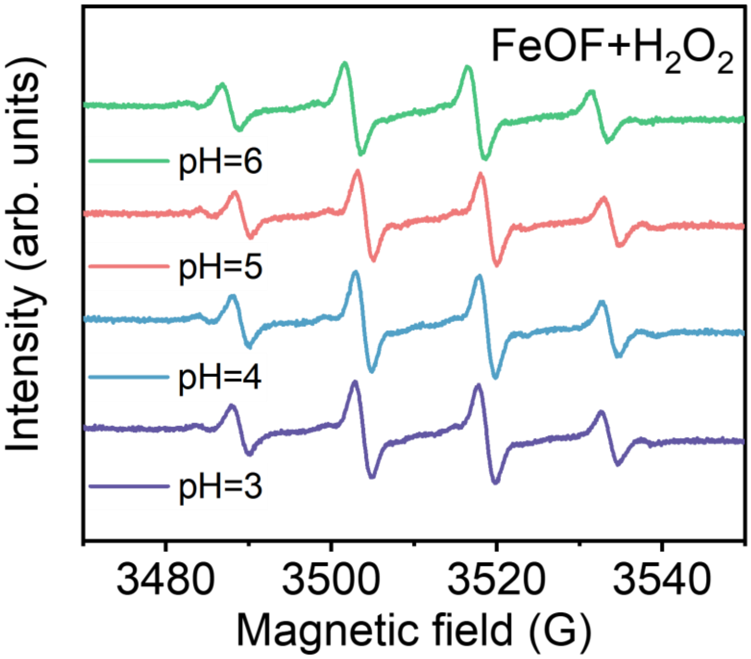

Supplementary Fig. 12. DMPO-•OH signals in EPR spectra of FeOF/H_2O_2 system at various pH values. Reaction conditions: [H_2O_2] = 10 mM, [catalyst] = 1.0 g L^{-1}, [DMPO] = 10 mM, pH = 7.0, Temperature = 20 °C.

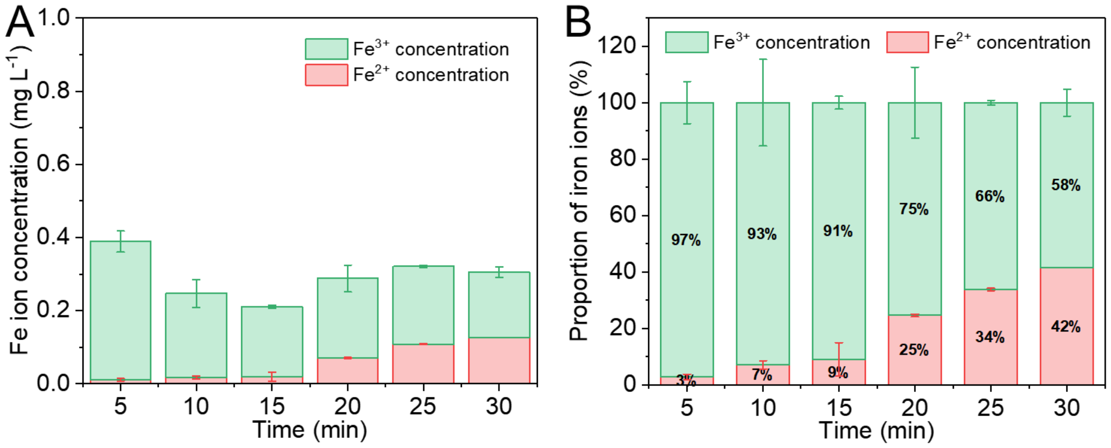

Supplementary Fig. 13. Concentration (A) and proportion (B) of leached Fe^{3+} and Fe^{2+} ions in the FeOF/H_2O_2 system. Source data are provided as a Source Data file.

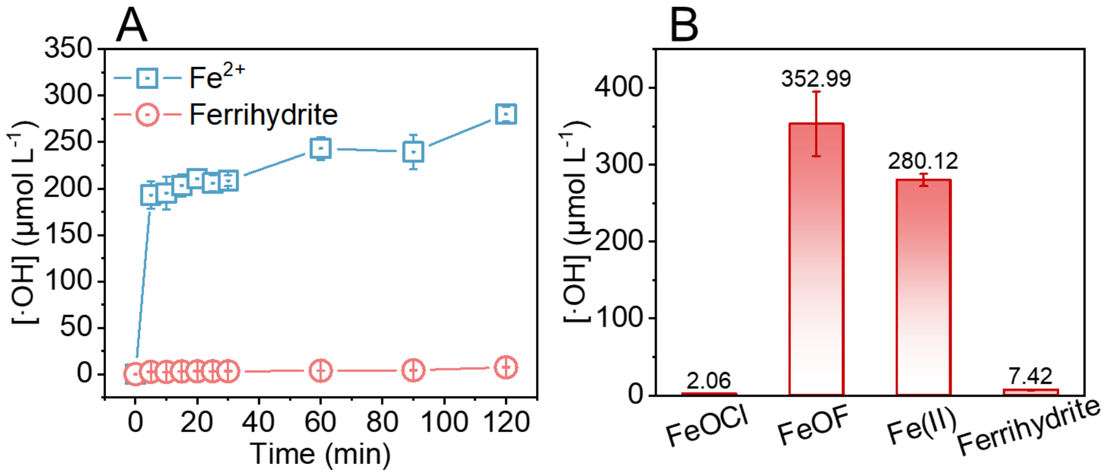

Supplementary Fig. 14. (A) Accumulated •OH generation versus reaction time in neutral ferrihydrite and conventional homogeneous Fenton systems. (B) Comparison of •OH generation in the FeOF, FeOCl, ferrihydrite and Fe^{2+} systems. Reaction conditions: [H_2O_2] = 10 mM, [catalyst] (except Fe^{2+}) = 0.1 g L^{-1}, [Fe^{2+}] = 1.10 mM, [Coumarin] = 10 mM, pH = 7.0, Temperature = 20 °C. Source data are provided as a Source Data file.

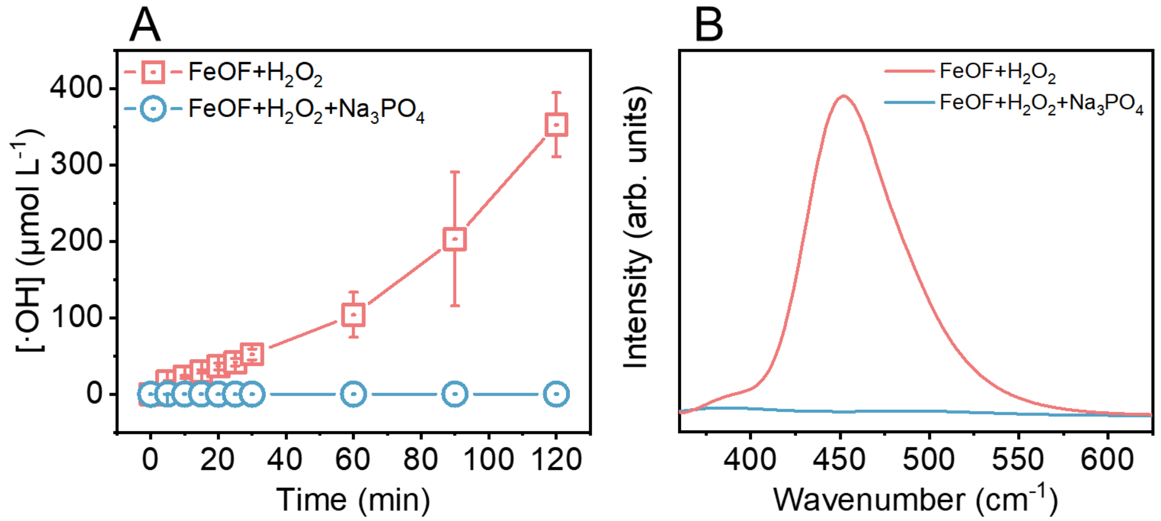

Supplementary Fig. 15. Effect of Na_3PO_4 on •OH generation during H_2O_2 activation by FeOF. (A) Accumulated •OH generation versus reaction time and (B) fluorescence spectra of 7-HC in the FeOF/H_2O_2 system with and without Na_3PO_4 addition. Reaction conditions: [H_2O_2] = 10 mM, [catalyst] = 0.1 g L^{-1}, [Coumarin] = 10 mM, [Na_3PO_4] = 2.0 g L^{-1} (if used), pH = 7.0, Temperature = 20 °C.

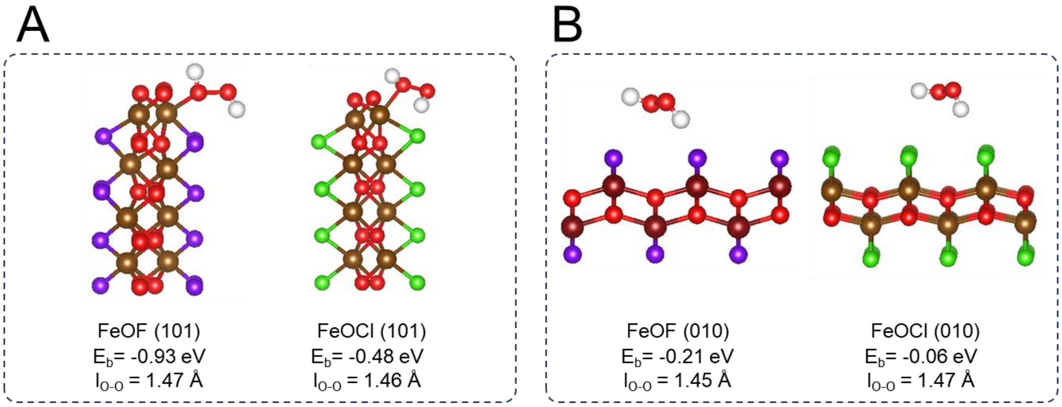

Supplementary Fig. 16. Optimized adsorption conformations of H_2O_2 at Fe site on the (101) and (010) facets of FeOF (A) and FeOCl (B). The brown, red, violet, and green balls represent the Fe, O, F, and Cl atoms, respectively.

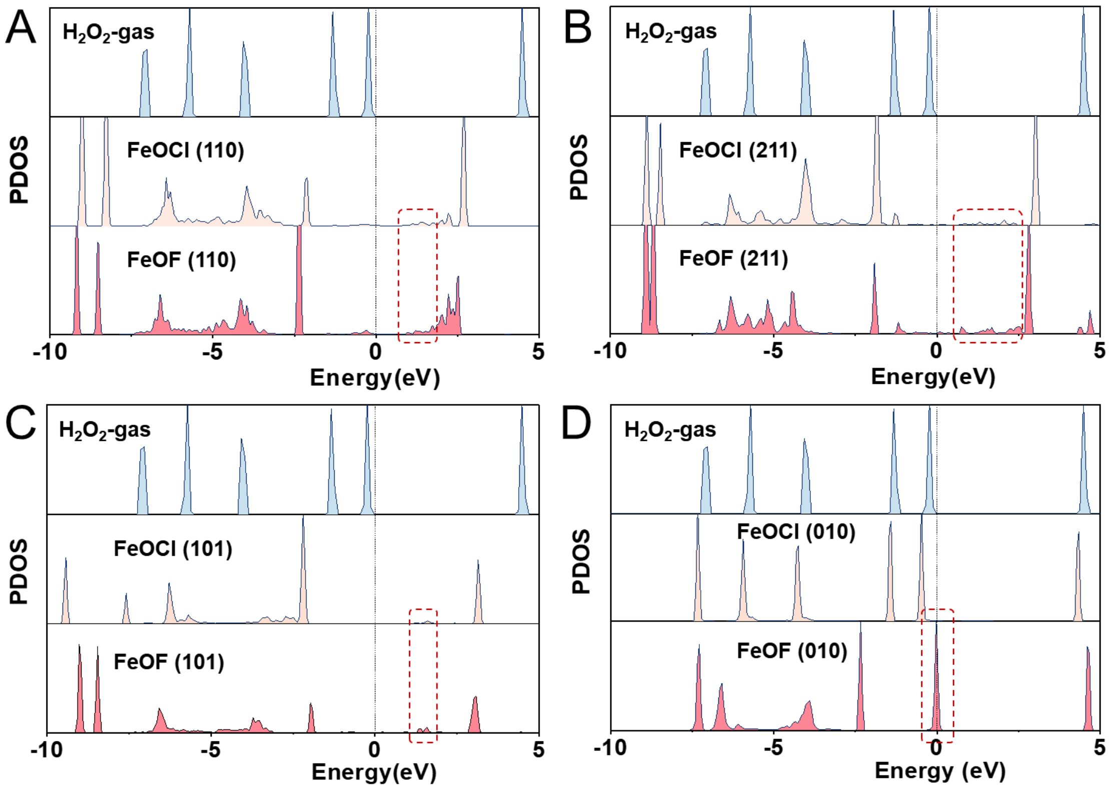

Supplementary Fig. 17. Partial density of states (PDOS) of H_2O_2 molecules before and after adsorption onto (110) (A), (211) (B), (101) (C), and (010) (D) facets of FeOF and FeOCl.

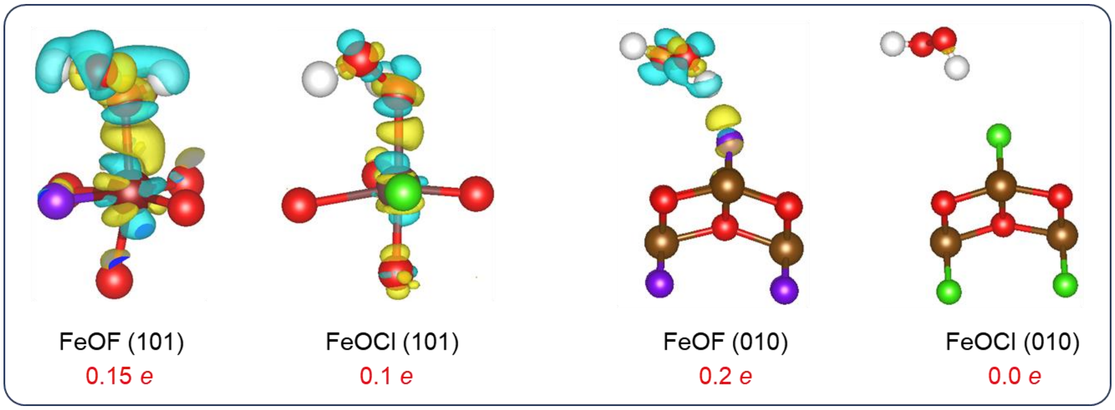

Supplementary Fig. 18. Charge density difference for H_2O_2 adsorption at Fe site on the (101) and (010) facets of FeOF and FeOCl. Yellow and blue contours represent electron accumulation and deletion, respectively. The isosurface contour is 0.003 e/Å^3. The brown, red, violet, and green balls represent the Fe, O, F, and Cl atoms, respectively.

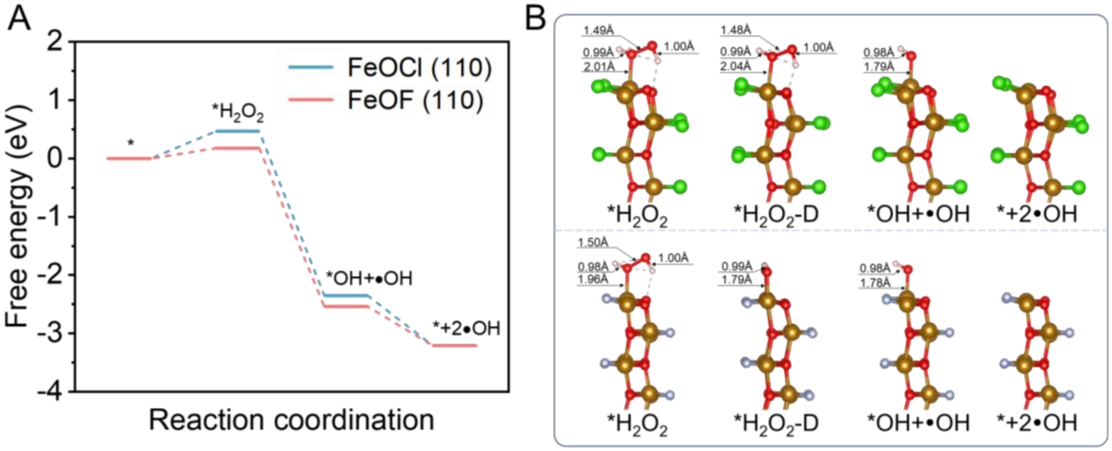

Supplementary Fig. 19. (A) Calculated free energy profiles for H_2O_2 activation and •OH formation on (110) facet of FeOF and FeOCl. (B) The corresponding structures of reaction intermediates as well as the coordination environment evolution of iron site on (110) facet for the generation of •OH. The brown, red, gray, and green balls represent the Fe, O, F, and Cl atoms, respectively. At initial step (i), the iron site on the surface of (110) facet shows pentahedral coordination structure with 3 oxygen atoms and 2 fluorine or chlorine atoms. The coordination number of iron site increases by one upon H_2O_2 adsorption and returns to original ones after H_2O_2 activation and •OH dissociation.

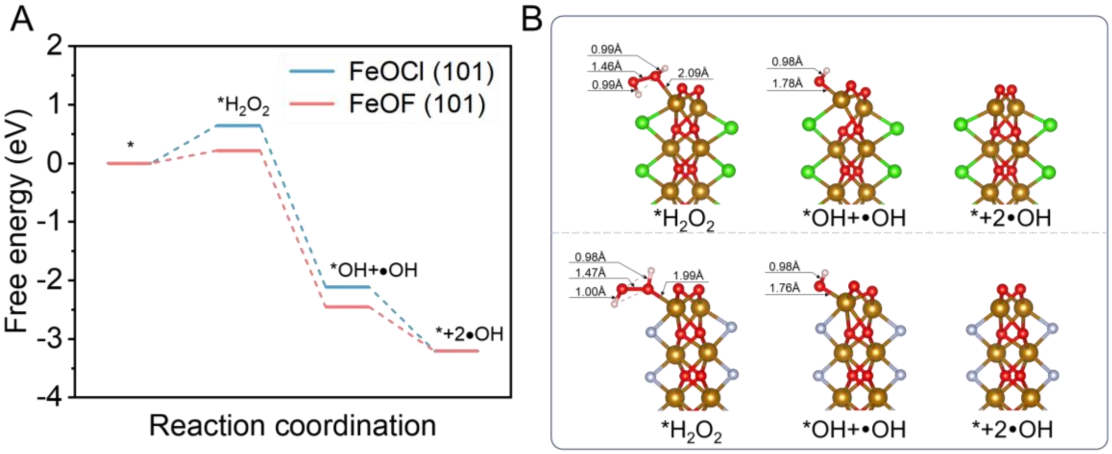

Supplementary Fig. 20. (A) Calculated free energy profiles for H_2O_2 activation and •OH formation on (101) facet of FeOF and FeOCl. (B) The corresponding structures of reaction intermediates as well as the coordination environment evolution of iron site on (101) facet for the generation of •OH. The brown, red, gray, and green balls represent the Fe, O, F, and Cl atoms, respectively. At initial step (i), the iron site on the surface of (101) facet shows pentahedral coordination structure with 3 oxygen atoms and 2 fluorine or chlorine atoms. The coordination number of iron site increases by one upon H_2O_2 adsorption and returns to original ones after H_2O_2 activation and •OH dissociation.

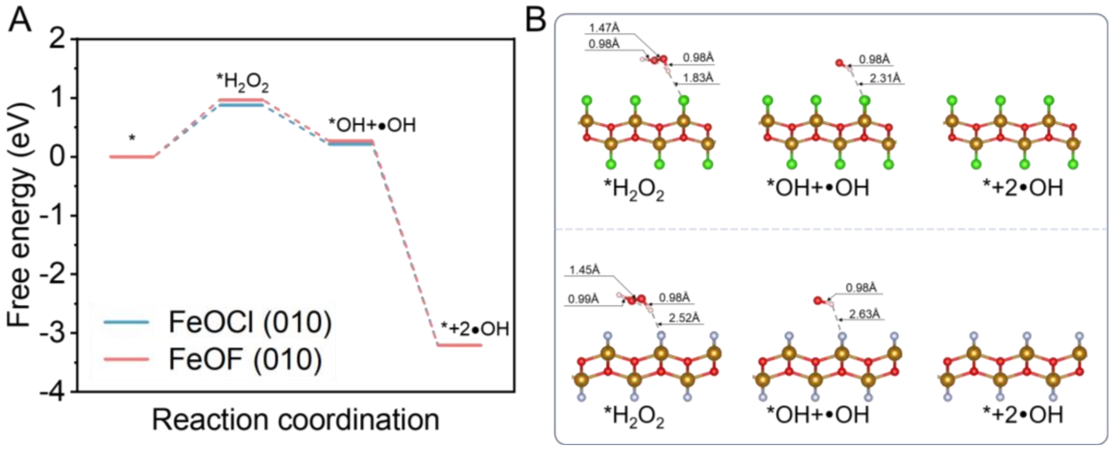

Supplementary Fig. 21. (A) Calculated free energy profiles for H_2O_2 activation and •OH formation on (010) facet of FeOF and FeOCl. (B) The corresponding structures of reaction intermediates as well as the coordination environment evolution of iron site on (010) facet for the generation of •OH. The brown, red, gray, and green balls represent the Fe, O, F, and Cl atoms, respectively. At initial step (i), the iron site on the surface of (010) facet shows saturated coordination structure with 4 oxygen atoms and 2 fluorine or chlorine atoms. The coordination number of iron site remains unchanged during Fenton reaction. The highest energy barrier for H_2O_2 adsorption indicates that H_2O_2 is hard to be adsorbed and activated by iron sites, which explains the inferior catalytic performance of (010) facets.

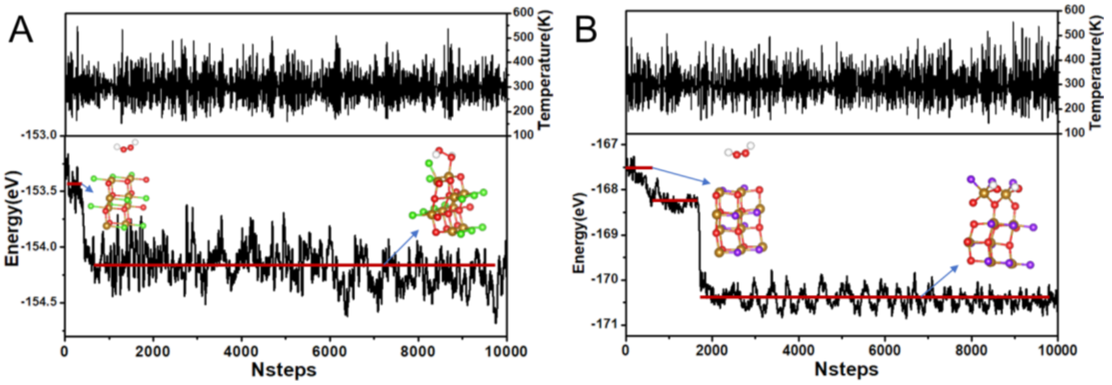

Supplementary Fig. 22. Evolution of temperature and energy during the ab initio molecular dynamics (MD) simulation for H_2O_2 activation onto FeOCl (A) and FeOF (B). The brown, red, green, blue, and white balls denote Fe, O, C, Cl, F, and H atoms, respectively.

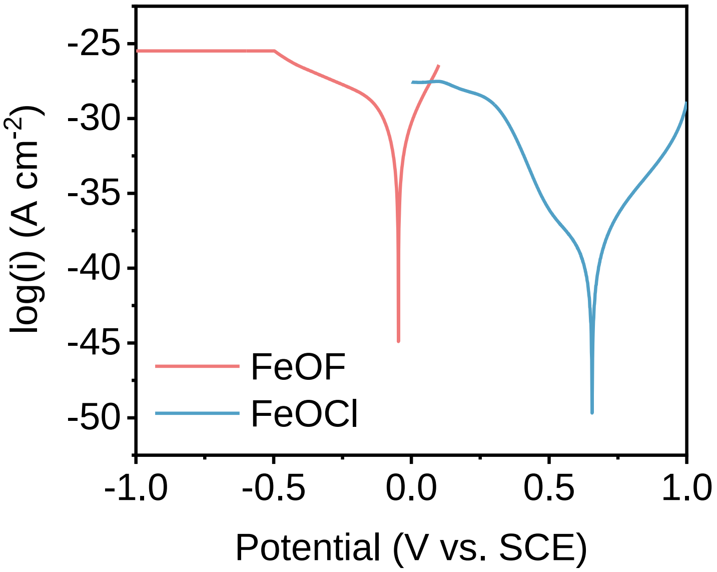

Supplementary Fig. 23. Tafel polarization curves of FeOF and FeOCl.

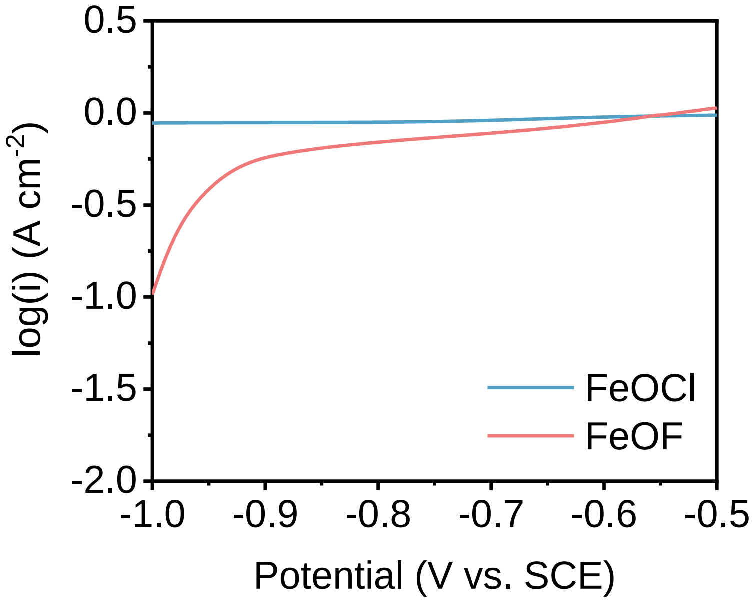

Supplementary Fig. 24. Linear sweep voltammetry (LSV) plots of FeOF and FeOCl.

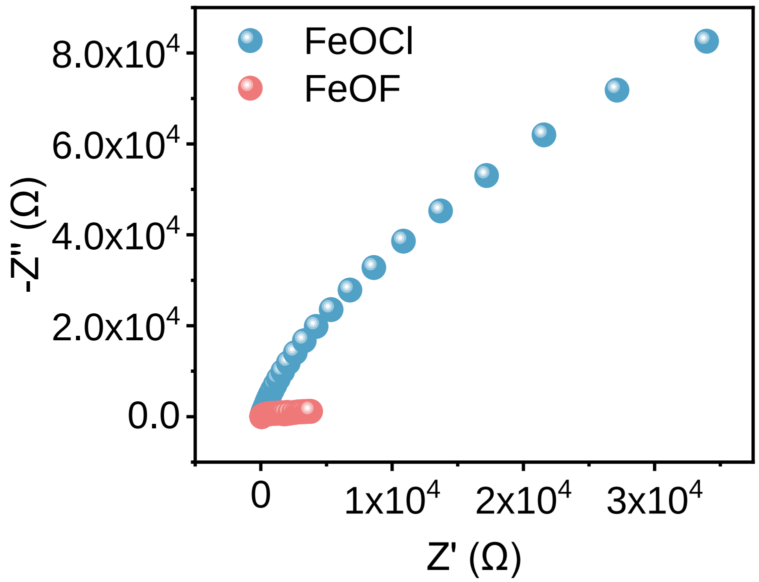

Supplementary Fig. 25. EIS spectra of FeOF and FeOCl.

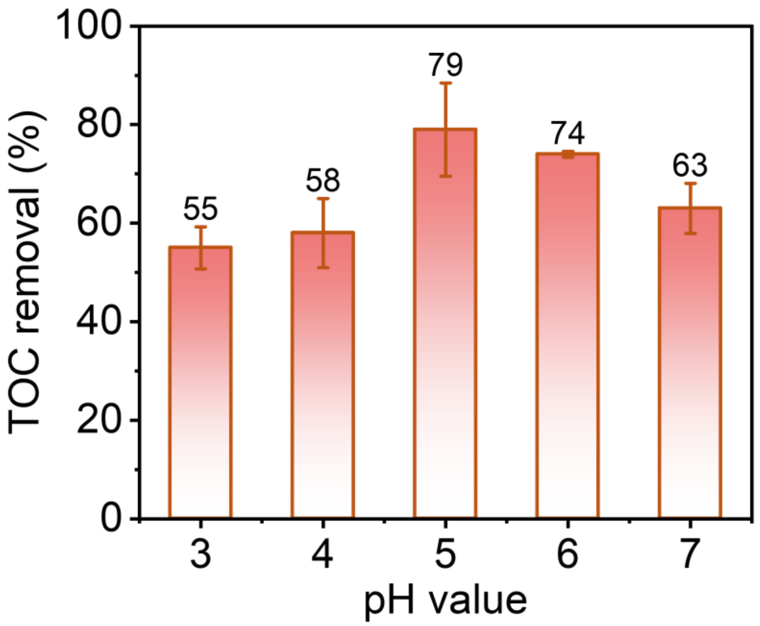

Supplementary Fig. 26. Effects of pH value on TOC removal by the FeOF/H_2O_2 system. Reaction conditions: [H_2O_2] = 10 mM, [catalyst] = 0.1 g L^{-1}, [4-NP] = 20 mg L^{-1}, pH = 7.0, Temperature = 20 °C. Source data are provided as a Source Data file.

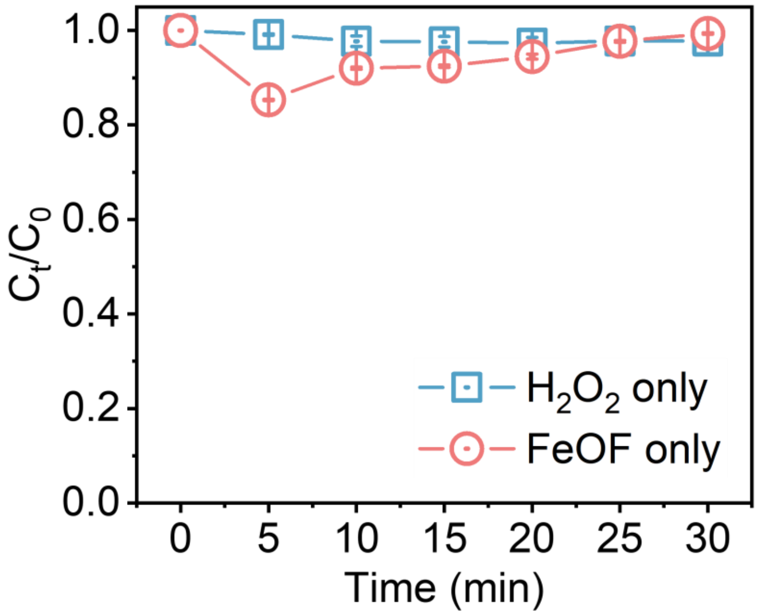

Supplementary Fig. 27. Degradation rate of 4-NP by individual H_2O_2 or FeOF system. Reaction conditions: [H_2O_2] = 10 mM (if used), [catalyst] = 0.1 g L^{-1} (if used), [4-NP] = 20 mg L^{-1}, pH = 7.0, Temperature = 20 °C. Source data are provided as a Source Data file.

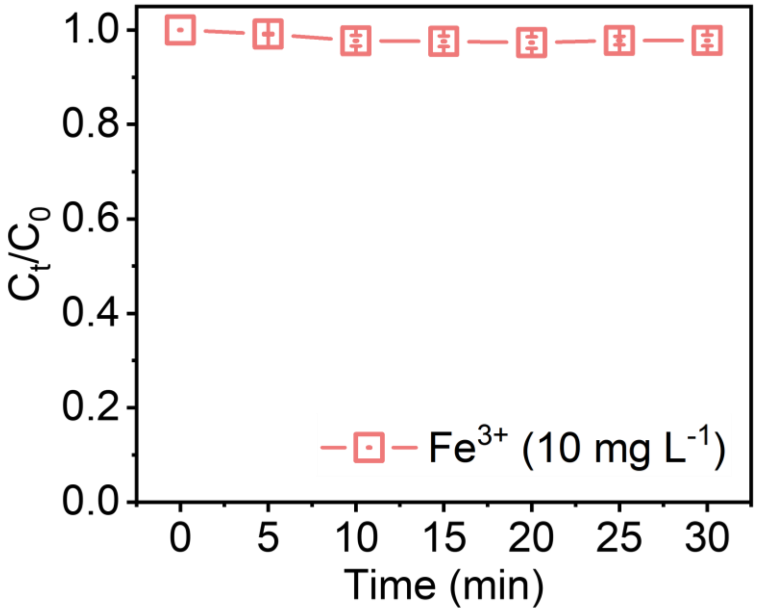

Supplementary Fig. 28. H_2O_2 activation performance of free Fe^{3+} ions with much higher concentration than the leachate of FeOF/H_2O_2 system. Reaction conditions: [H_2O_2] = 10 mM, [Fe^{3+}] = 10 mg L^{-1}, [4-NP] = 20 mg L^{-1}, pH = 7.0, Temperature = 20 °C.

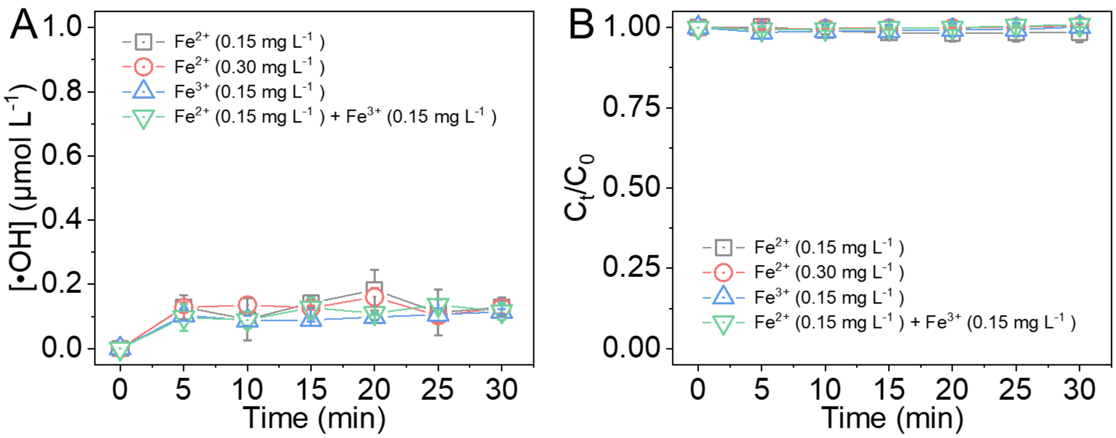

Supplementary Fig. 29. Accumulated •OH generation (A) and 4-NP degradation (B) by H_2O_2 activation using Fe^{2+} or/and Fe^{3+} ions as the catalyst. Reaction conditions: [H_2O_2] = 10 mM, [Coumarin] = 10 mM (if used), [4-NP] = 20 mg L^{-1} (if used), pH = 7.0, Temperature = 20 °C. Source data are provided as a Source Data file.

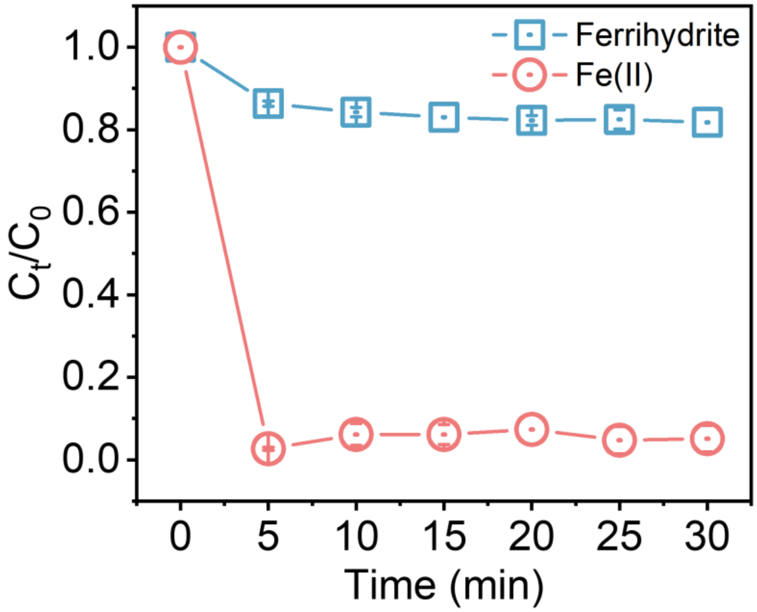

Supplementary Fig. 30. Degradation of 4-NP by ferrihydrite/H_2O_2 and conventional homogeneous Fe(II) systems. Reaction conditions: [H_2O_2] = 10 mM, [catalyst] = 0.1 g L^{-1}, [Fe^{2+}] = 1.10 mM, [4-NP] = 20 mg L^{-1}, pH = 7.0, Temperature = 20 °C. Source data are provided as a Source Data file.

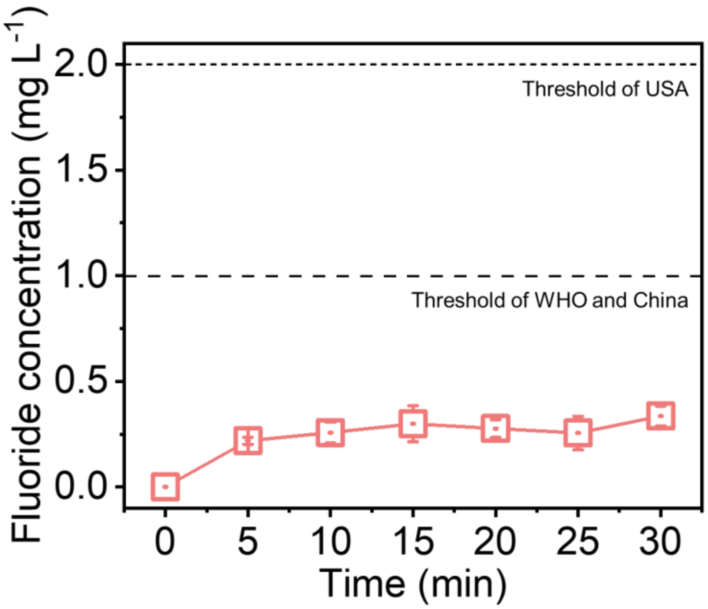

Supplementary Fig. 31. Concentration of fluoride leaching during Fenton reaction with FeOF as the catalyst. Reaction conditions: [H_2O_2] = 10 mM, [catalyst] = 0.1 g L^{-1}, [4-NP] = 20 mg L^{-1}, pH = 7.0, Temperature = 20 °C. Source data are provided as a Source Data file.

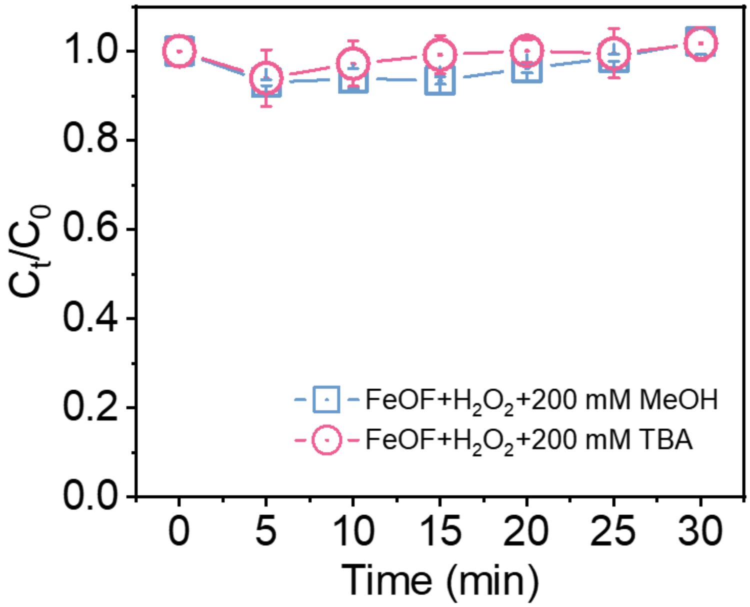

Supplementary Fig. 32. Quenching effect of high concentration TBA and methanol on the 4-NP degradation efficiency by FeOF. Reaction conditions: $[H_2O_2]$ = 10 mM, [catalyst] = 0.1 g L^{-1}, [4-NP] = 20 mg L^{-1}, [MeOH] = [TBA] = 200 mM, pH = 7.0, Temperature = 20 °C.

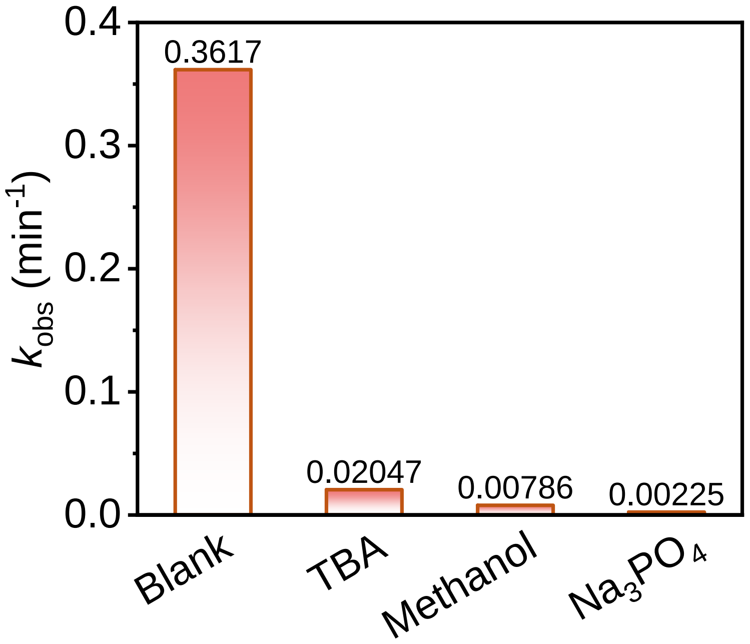

Supplementary Fig. 33. Quenching or inhibition effect of TBA, methanol, and Na_3PO_4 on the observed rate constant of 4-NP degradation by FeOF catalyst.

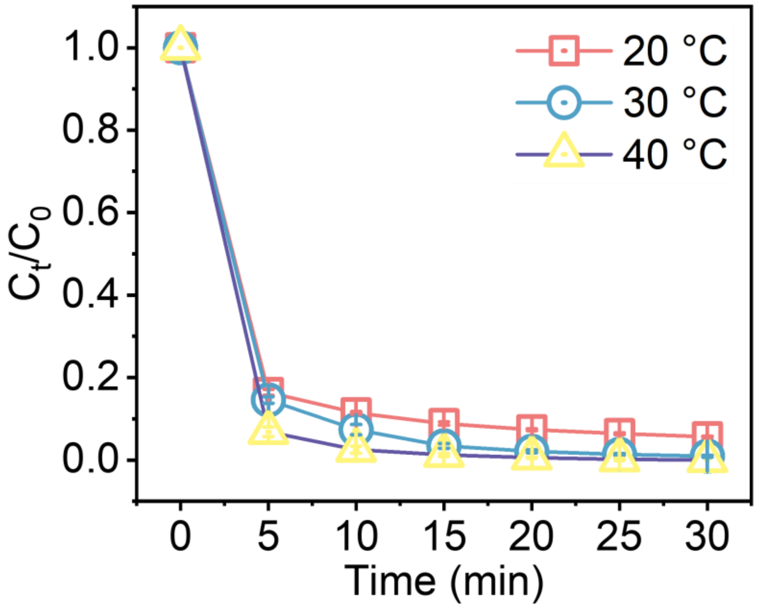

Supplementary Fig. 34. Effects of reaction temperature on 4-NP degradation. Reaction conditions: $[H_2O_2]$ = 10 mM, [catalyst] = 0.1 g L^{-1}, [4-NP] = 20 mg L^{-1}, pH = 7.0. Source data are provided as a Source Data file.

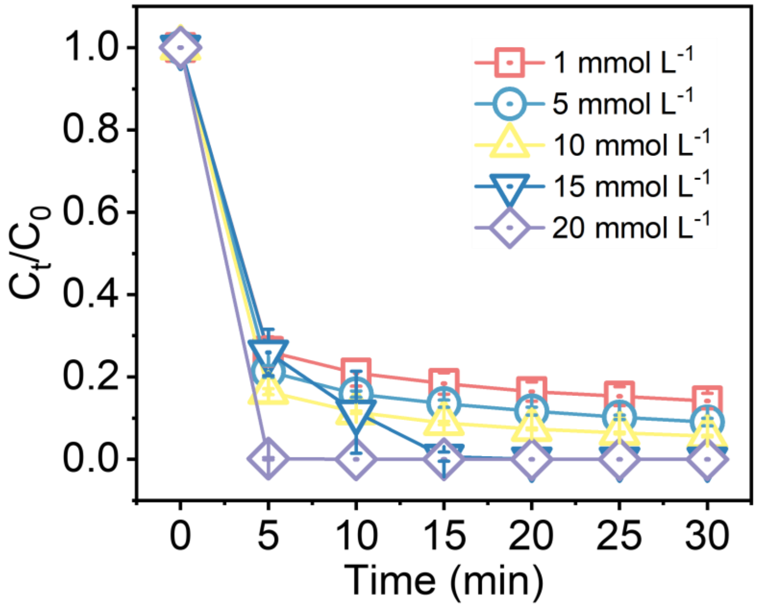

Supplementary Fig. 35. Effects of H_2O_2 dosage on 4-NP degradation. Reaction conditions: [catalyst] = 0.1 g L^{-1}, [4-NP] = 20 mg L^{-1}, pH = 7.0, Temperature = 20 °C. Source data are provided as a Source Data file.

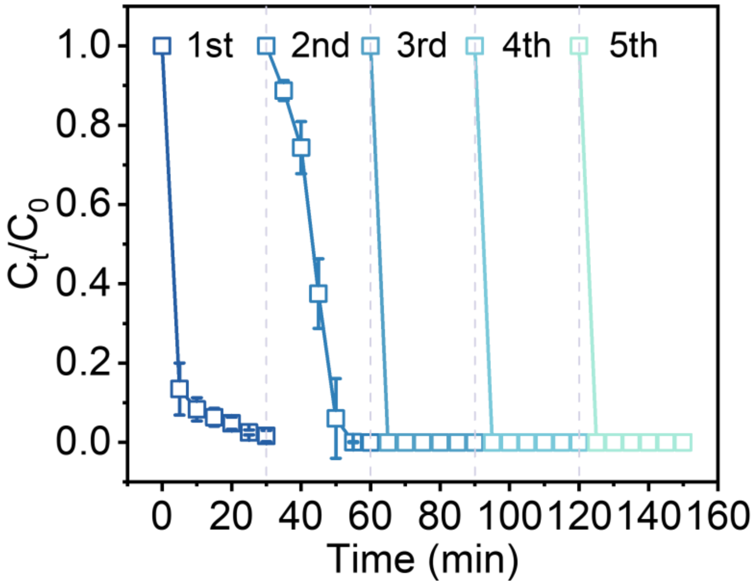

Supplementary Fig. 36. Successive recycle tests with the addition of both 4-NP and H_2O_2 after each cycle. Reaction conditions: [H_2O_2] = 10 mM, [catalyst] = 0.1 g L^{-1}, [4-NP] = 20 mg L^{-1}, pH = 7.0, Temperature = 20 °C.

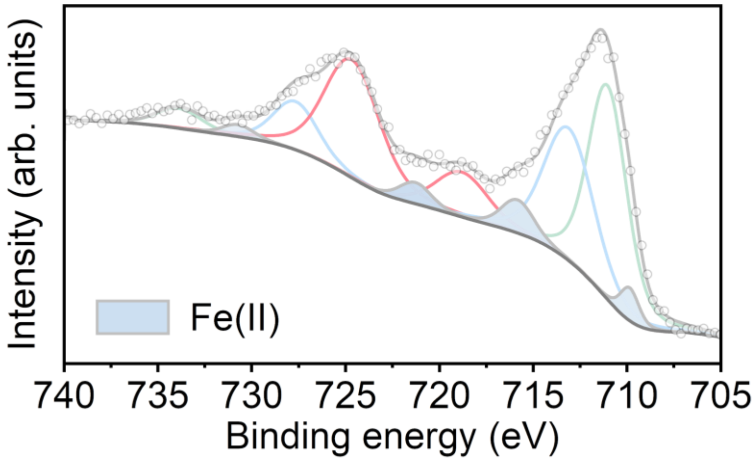

Supplementary Fig. 37. High-resolution Fe 2p XPS spectrum of the FeOF after recycling.

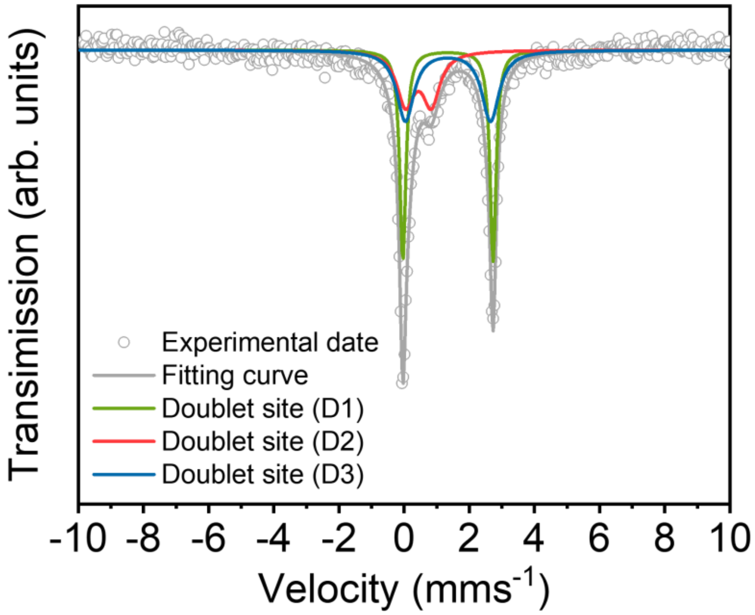

Supplementary Fig. 38. ^{57}Fe Mössbauer spectrum of the FeOF after recycling.

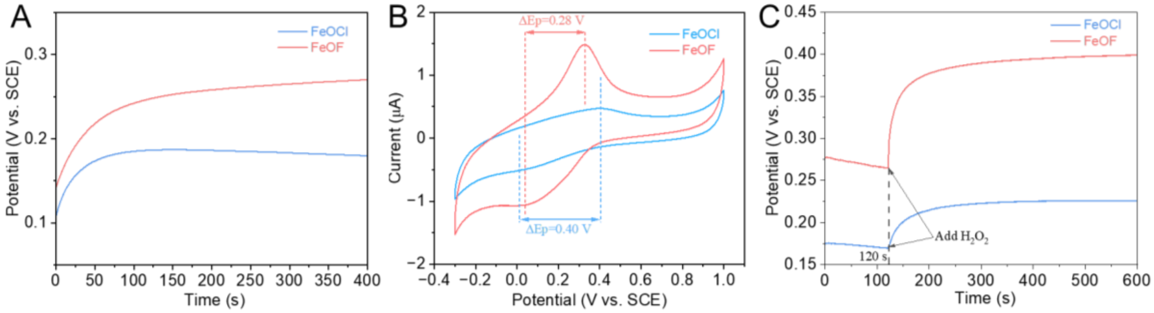

Supplementary Fig. 39. (A) Open-circuit potential curves measurements, (B) cyclic voltammetry measurements, and (C) open-circuit potential curves with the in-situ addition of H_2O_2 on FeOF and FeOCl coated glassy carbon electrode.

Note: The open-circuit potential displayed in Supplementary Fig. 39A highlights a higher potential of Fe(III) in FeOF (0.142 V) than that in FeOCl (0.108 V), affirming the stronger activity to produce Fe(II) that results in the stronger oxidation capacity of the FeOF-catalyzed Fenton system (13). As shown in Supplementary Fig. 39B, obvious oxidation and reduction peaks appeared at 0.04 and 0.32 V with FeOF, implying the excellent reversibility of the Fe(II)/Fe(III) redox process. The fluorine coordination also leads to a decrease in peak-to-peak separation (ΔE_p) from 0.40 V to 0.28 V, indicating the improved Fe(II)/Fe(III) redox capability of FeOF (14). Furthermore, the in-situ electrode potential analysis was performed to investigate the redox cycle of the iron center during the Fenton reaction. The addition of H_2O_2 triggers the increase in open-circuit potentials for both FeOF and FeOCl while the former exhibits a more substantial increment (Supplementary Fig. 39C), which further elucidates the more facile reversibility of the Fe(II)/Fe(III) cycle with fluorine coordination (15). In addition, we found that the proportion of leaching Fe^{2+} ions increased from 3% to 42% (Supplementary Fig. 13), indicating the enhancement of Fe(II)/Fe(III) redox cycle.

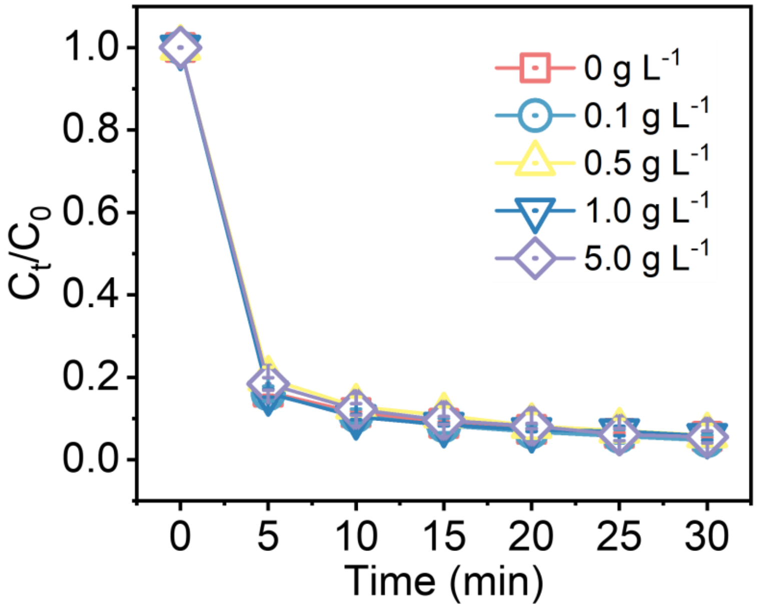

Supplementary Fig. 40. Effects of NaCl dosage on 4-NP degradation. Reaction conditions: [H_2O_2] = 10 mM, [catalyst] = 0.1 g L^{-1}, [4-NP] = 20 mg L^{-1}, [NaCl] = 0~5.0 g L^{-1}, pH = 7.0, Temperature = 20 °C. Source data are provided as a Source Data file.

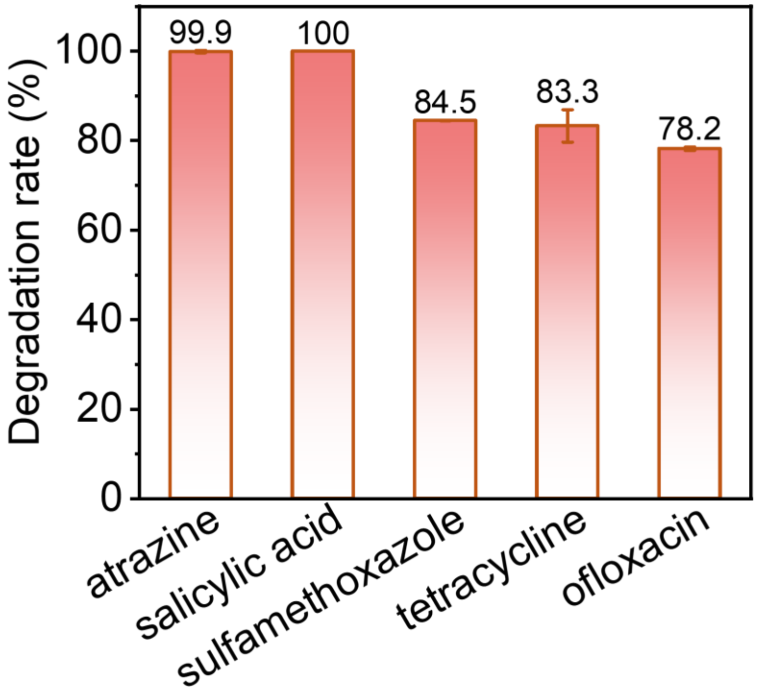

Supplementary Fig. 41. Degradation rate of screened organic pollutants by FeOF/H_2O_2 system. Source data are provided as a Source Data file.

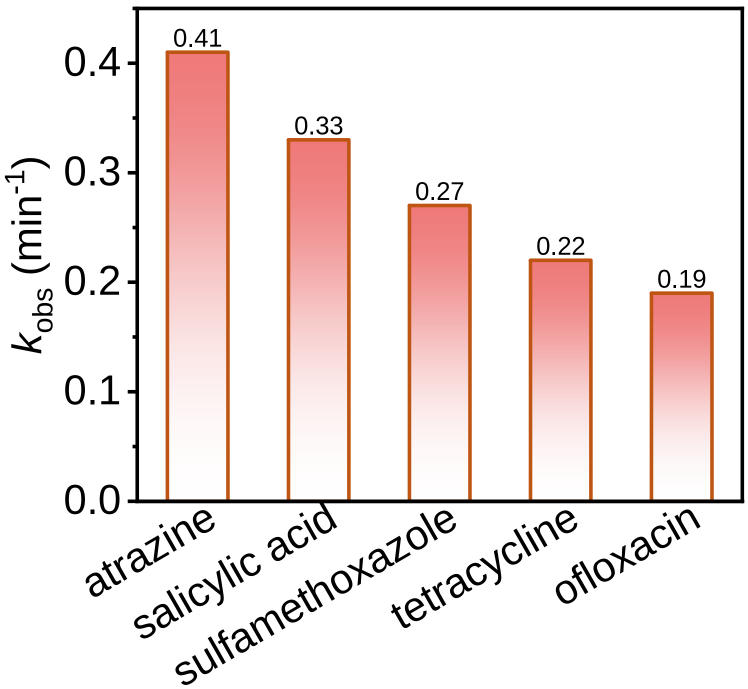

Supplementary Fig. 42. k_{obs} values for the screened organic pollutants degradation by FeOF/H_2O_2 system.

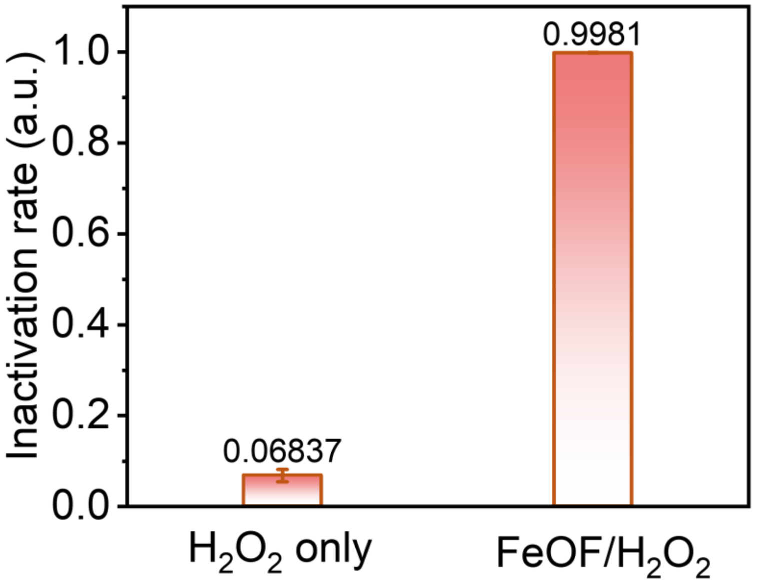

Supplementary Fig. 43. Bacterial inactivation performance of the FeOF/H_2O_2 system. Reaction conditions: [H_2O_2] = 10 mM, [catalyst] = 0.1 g L^{-1}, [*E. coli*] = 10^7 CFU mL^{-1}, pH = 7.0, Temperature = 20 °C.

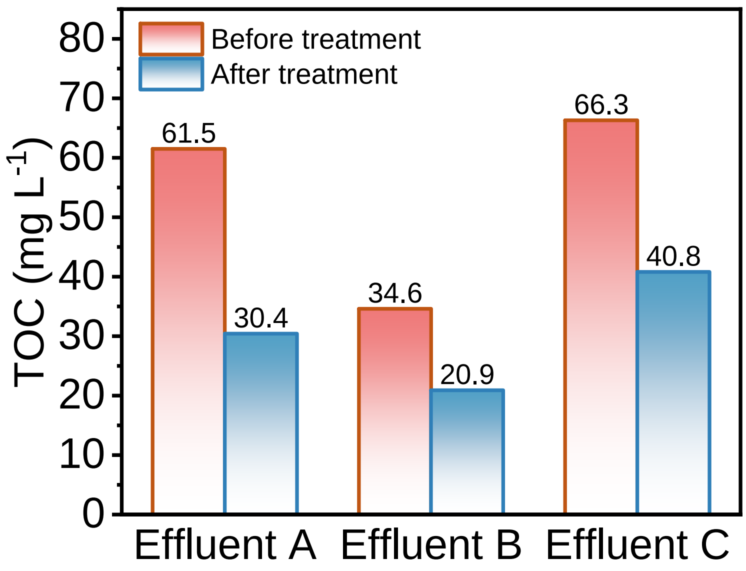

Supplementary Fig. 44. TOC value change of collected wastewaters before and after treatment by FeOF/H_2O_2 system. Reaction conditions: [H_2O_2] = 10 mM, [catalyst] = 0.1 g L^{-1}, pH = 7.0, Temperature = 20 °C.

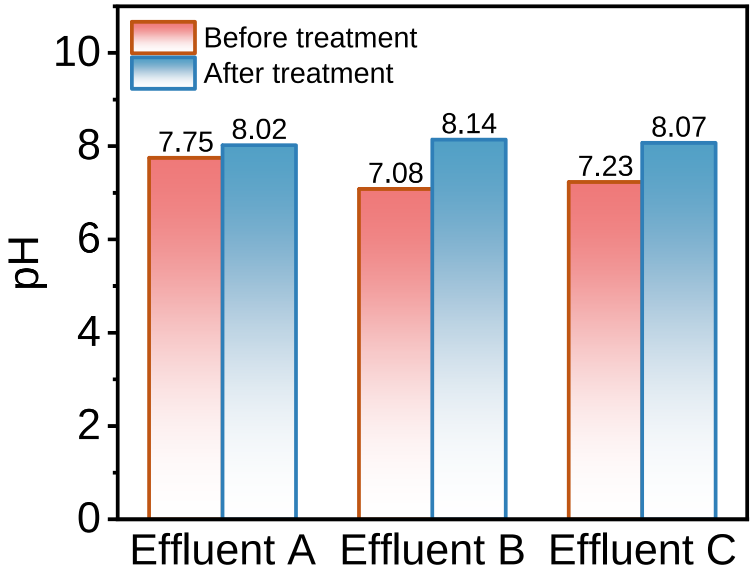

Supplementary Fig. 45. pH value change of collected wastewaters before and after treatment by FeOF/H_2O_2 system.

Supplementary Tables

Supplementary Table 1. Information of porous architecture of FeOF and FeOCl catalysts.

Sample	S_{BET} (m^2 g^{-1})	Average Pore diameter (nm)	Pore volume (cm^3 g^{-1})
FeOF	18.572	18.093	0.084
FeOCl	9.259	43.742	0.101

Supplementary Table 2. Mössbauer fitting parameters and relative contents of five Fe components as estimated from the relative absorption areas of the fitting peaks in the spectrum for FeOF.

**Sample**	**Fitting Peaks**	**Fe species**	**IS[a] (mm s^{-1})**	**QS[b] (mm s^{-1})**	**Line Width (mm s^{-1})**	**Content (%)**
Raw FeOF	D1	F-(Fe(III)O_4)-F high-spin structure	0.38	0.99	0.42	54.0
	D2	F-(Fe(III)O_4) or F-(Fe(III)O_3)-F medium-spin structure	0.37	0.58	0.26	46.0
Spent FeOF	D1	F-(Fe(III)O_4)-F high-spin structure	1.34	2.77	0.22	37.7
	D2	F-(Fe(II)O_4) or F-(Fe(II)O_3)-F medium-spin structure	0.44	0.79	0.64	26.5
	D3	F-(Fe(III)O_4) or F-(Fe(III)O_3)-F medium-spin structure	1.34	2.61	0.64	35.9

[a] Isomer shift; [b] Quadrupole splitting.

Supplementary Table 3. EXAFS fitting parameters at the Fe K-edge for various samples.

Sample	Scattering pair	CN[a]	R(Å)[b]	σ^2 (Å^2)[c]	ΔE_0 (eV)[d]	R factor
FeOF	Fe-O	2.7	1.90	0.0072	-5.48	0.0226
	Fe-F	1.9	1.99	0.0080		
	Fe-Fe	3.6	2.96	0.0171		

[a] CN: coordination numbers; [b] R: bond distance; [c] σ^2: Debye-Waller factors; [d] ΔE_0: the inner potential correction. *R* factor: goodness of fit.

Supplementary Table 4. Summary of apparent rate constants for •OH generation by FeOF and FeOCl with and without normalized by specific surface area and the amount of Lewis acid sites.

Catalyst	Apparent rate constant for •OH generation, $K_{(\cdot OH)}$ (µmol L^{-1} min^{-1})	Apparent rate constant for •OH generation normalized by specific surface area, K_S (µmol L^{-1} min^{-1})	Apparent rate constant for •OH generation normalized by the amount of Lewis acid sites			
			Total	Weak	Medium	Strong
**FeOF**	2.94	31.7	194.2	-	346.1	442.3
**FeOCl**	0.017	0.371	3.2	7.8	5.6	85.9

Supplementary Table 5. Comparison of H_2O_2 activation performance of FeOF with other state-of-the-art Fenton catalysts at neutral or acid conditions.

**Sample**	**[·OH] (μmol L^{-1})**	**pH**	**H_2O_2→·OH selectivity***	**Catalyst Normalized Selectivity (g^{-1})**	**References**
FeOF	352.99	7	0.333	3.33	This work
FeOCl	2.06	7	0.000206	0.00206	
Fe(II)	280.12	3	0.0451	0.206	
Ferrihydrite	7.42	3	0.000742	0.00742	
La-Cu-Al	193.26	4	1.11	1.11	(16)
Cu_5/FeS_2	180	7	0.483	0.967	(17)
FeB	145.52	3	0.404	2.02	(18)
P-Cu-Al_2O_3	135.34	6.3	0.0607	0.0607	(19)
DBC-FeO_x	131.6	3.5	0.251	2.51	(20)
$CuFe_2O_4$	79.51	5	0.00795	0.00795	(21)
nano-Fe_2O_3	54	5	0.0054	0.0054	(22)
meso-$CuFe_2O_4$	53.81	3	0.00134	0.00448	(23)
Siderite	36.77	6	0.0483	0.0483	(24)
nano-Fe_3O_4	34.86	3	0.000871	0.00290	(23)
FeS_2	30	7	0.0534	0.107	(17)
MIL-53(Cu)	30	5	0.003	0.003	(22)
HS-$CuFe_2O_4$-σ	26.44	6.5	0.00203	0.00407	(25)
CQDs-Fe(III)	25.28	5	0.0505	2.81	(26)

Sample	[·OH] (μmol L^{-1})	pH	$H_2O_2 \rightarrow \cdot OH$ selectivity*	Catalyst Normalized Selectivity (g^{-1})	References
ZrO_2–Fe_2O_3	23.63	7	0.00236	0.00472	(27)
CD-COOFe(III)	13.95	4.5	0.0279	2.32	(28)
FeOCl	11	7	0.000733	0.00367	(29)
Fe_3O_4	10.5	4	0.525	0.525	(30)
$BiFeO_3$	0.66	4.5	0.000122	0.000122	(31)

* $H_2O_2 \rightarrow \cdot OH$ selectivity = H_2O_2 utilization efficiency = $[\cdot OH]_t/\Delta[H_2O_2]$*100%. By detection with a titanium (IV) spectrophotometric method, we observed 10.6% of H_2O_2 was consumed in the FeOF/H_2O_2 system.

Supplementary Table 6. Information (*i.e.*, E_b, $I_{O\text{-}O}$, and charge transfer) for the H_2O_2 adsorption onto various facets of FeOF and FeOCl.

Sample	Facet	E_b (eV)	$I_{O\text{-}O}$ (Å)	Charge transfer ΔQ_{Fe} (e^-)
FeOF	(101)	-0.93	1.47	0.15
	(110)	-0.84	1.50	0.15
	(211)	-0.91	1.48	0.18
	(010)	-0.21	1.45	0.20
FeOCl	(101)	-0.48	1.46	0.10
	(110)	-0.78	1.49	0.10
	(211)	-0.18	1.47	0.10
	(010)	-0.06	1.47	0.00

Supplementary Table 7. Reaction energy of the intermediates in the H_2O_2 activation process by different facets of FeOF and FeOCl catalysts.

**Sample**	**Facet**	**$\Delta E_{(*\rightarrow *H2O2)}$ (eV)**	**$\Delta E_{(*H2O2\rightarrow *OH+\bullet OH)}$ (eV)**	**$\Delta E_{(*OH+\bullet OH\rightarrow *+2\bullet OH)}$ (eV)**
FeOF	(101)	0.22	-2.67	-0.75
	(110)	0.18	-2.72	-0.67
	(211)	-0.14	-2.72	-0.34
	(010)	0.96	-0.69	-3.48
FeOCl	(101)	0.64	-2.76	-1.09
	(110)	0.46	-2.82	-0.86
	(211)	0.54	-3.30	-0.45
	(010)	0.87	-0.66	-3.42

Supplementary References

1. H. Wang *et al.*, Modulating inherent lewis acidity at the intergrowth interface of mortise-tenon zeolite catalyst. *Nat. Commun.* **13** 2924 (2022).
2. C. A. Emeis, Determination of Integrated Molar Extinction Coefficients for IR Absorption Bands of Pyridine Adsorbed on Solid Acid Catalysts. *J. Catal.* **141** 347-354 (1993).
3. F. Mo *et al.*, The optimized Fenton- like activity of Fe single- atom sites by Fe atomic clusters-mediated electronic configuration modulation. *Proc. Natl. Acad. Sci. USA* **120** e2300281120 (2023).
4. G. Kresse *et al.*, Efficient iterative schemes for ab initio total-energy calculations using a plane-wave basis set. *Phys. Rev. B* **54**, 11169-11186 (1996).
5. J. P. Perdew *et al.*, Atoms, molecules, solids, and surfaces: Applications of the generalized gradient approximation for exchange and correlation. *Phys. Rev. B* **46**, 6671-6687 (1992).
6. P. E. Blöchl, Projector augmented-wave method. *Phys. Rev. B* **50**, 17953-17979 (1994).
7. J. P. Perdew *et al.*, Accurate and simple analytic representation of the electron-gas correlation energy. *Phys. Rev. B* **45**, 13244-13249 (1992).
8. S. Grimme, Semiempirical GGA-type density functional constructed with a long-range dispersion correction. *J. Comput. Chem.* **27**, 1787-1799 (2006).
9. X. Xu *et al.*, Revealing *OOH key intermediates and regulating H2O2 photoactivation by surface relaxation of Fenton-like catalysts. *Proc. Natl. Acad. Sci. USA* **119** e2205562119 (2022).
10. A. F. de Faria *et al.*, Antimicrobial Electrospun Biopolymer Nanofiber Mats Functionalized with Graphene Oxide–Silver Nanocomposites. *ACS Appl. Mat. Interfaces* **7**, 12751-12759 (2015).
11. F. Perreault *et al.*, Antimicrobial Properties of Graphene Oxide Nanosheets: Why Size Matters. *ACS Nano* **9**, 7226-7236 (2015).
12. L. M. Gilbertson *et al.*, Shape-Dependent Surface Reactivity and Antimicrobial Activity of Nano-Cupric Oxide. *Environ. Sci. Technol.* **50**, 3975-3984 (2016).
13. W. Tan *et al.*, Peroxymonosulfate activated with waste battery-based Mn-Fe oxides for pollutant removal: Electron transfer mechanism, selective oxidation and LFER analysis. *Chem. Eng. J.* **394** 124864 (2020).
14. M. Liu *et al.*, Accelerated Fe^{2+} Regeneration in an Effective Electro-Fenton Process by Boosting Internal Electron Transfer to a Nitrogen-Conjugated Fe(III) Complex. *Environ. Sci. Technol.* **55**, 6042-6051 (2021).
15. S.-C. Mei *et al.*, Heterogeneous Fenton water purification catalyzed by iron phosphide (FeP). *Water Res.* **241**, 120151-120151 (2023).
16. X. Zhang *et al.*, Enhanced •OH generation and pollutants removal by framework Cu doped $LaAlO_3/Al_2O_3$. *J Hazard Mater* **431**, 128578 (2022).
17. C. Ling *et al.*, Atomic-Layered Cu_5 Nanoclusters on FeS_2 with Dual Catalytic Sites for Efficient and Selective H_2O_2 Activation. *Angew. Chem. Int. Ed.* **61**, e202200670 (2022).
18. Y. Zhang *et al.*, Iron boride boosted Fenton oxidation: Boron species induced sustainable Fe(III)/Fe(II) redox couple. *J. Hazard. Mater.* **443**, 130386 (2023).

19. L. Li *et al.*, More octahedral Cu^+ and surface acid sites in uniformly porous Cu-Al_2O_3 for enhanced Fenton catalytic performances. *J. Hazard. Mater.* **406**, 124739 (2021).
20. H. Yu *et al.*, Facile preparation of coprecipitates between iron oxides and dissolved organic matter for efficient Fenton-like degradation of norfloxacin. *J. Hazard. Mater.* **444**, 130394 (2023).
21. Y. Ren *et al.*, An in-situ strategy to analyze multi-effect catalysis in iron-copper bimetals catalyzed Fenton-like processes. *Appl. Catal. B: Environ.* **299** 120697 (2021).
22. Y. Ren *et al.*, Enhancing the Fenton-like Catalytic Activity of nFe_2O_3 by MIL-53(Cu) Support: A Mechanistic Investigation. *Environ. Sci. Technol.* **54**, 5258-5267 (2020).
23. Y. Wang *et al.*, Magnetic ordered mesoporous copper ferrite as a heterogeneous Fenton catalyst for the degradation of imidacloprid. *Appl. Catal. B: Environ.* **147**, 534-545 (2014).
24. F. Sun *et al.*, A quantitative analysis of hydroxyl radical generation as H_2O_2 encounters siderite: Kinetics and effect of parameters. *Appl. Geochem.* **126** 104893 (2021).
25. R.-R. Ding *et al.*, Oxygen vacancy on hollow sphere $CuFe_2O_4$ as an efficient Fenton-like catalysis for organic pollutant degradation over a wide pH range. *Appl. Catal. B: Environ.* **291** 120069 (2021).
26. T. Zhang *et al.*, Overcoming Acidic H_2O_2/Fe(II/III) Redox-Induced Low H_2O_2 Utilization Efficiency by Carbon Quantum Dots Fenton-like Catalysis. *Environ. Sci. Technol.* **56**, 2617-2625 (2022).
27. Y. Yin *et al.*, Exploring the mechanism of ZrO_2 structure features on H_2O_2 activation in Zr–Fe bimetallic catalyst. *Appl. Catal. B: Environ.* **299** 120685 (2021).
28. T. Zhang *et al.*, Homogeneous Carbon Dot-Anchored Fe(III) Catalysts with Self-Regulated Proton Transfer for Recyclable Fenton Chemistry. *JACS Au* **3** 516-525 (2023).
29. M. Sun *et al.*, Reinventing Fenton Chemistry: Iron Oxychloride Nanosheet for pH-Insensitive H_2O_2 Activation. *Environ. Sci. Technol. Lett.* **5**, 186-191 (2018).
30. H. Sun *et al.*, Ascorbic acid promoted magnetite Fenton degradation of alachlor: Mechanistic insights and kinetic modeling. *Appl. Catal. B: Environ.* **267** 118383 (2020).
31. Q.-Q. Huang *et al*, Ligand-assisted heterogeneous catalytic H_2O_2 activation for pollutant degradation: The trade-off between coordination site passivation and adjacent site activation. *Appl. Catal. B: Environ.* **330** 122592 (2023).
